# The *Nitrosopumilus maritimus* CdvB, but Not FtsZ, Assembles into Polymers

**DOI:** 10.1155/2013/104147

**Published:** 2013-06-02

**Authors:** Kian-Hong Ng, Vinayaka Srinivas, Ramanujam Srinivasan, Mohan Balasubramanian

**Affiliations:** ^1^Cell Division Laboratory, Temasek Life Sciences Laboratory, 1 Research Link, National University of Singapore, Singapore 117604; ^2^Department of Biological Sciences, National University of Singapore, 14 Science Drive 4, Singapore 117543; ^3^Mechanobiology Institute, National University of Singapore, 5A Engineering Drive 1, Singapore 117411

## Abstract

Euryarchaeota and Crenarchaeota are two major phyla of archaea which use distinct molecular apparatuses for cell division. Euryarchaea make use of the tubulin-related protein FtsZ, while Crenarchaea, which appear to lack functional FtsZ, employ the Cdv (cell division) components to divide. Ammonia oxidizing archaeon (AOA) *Nitrosopumilus maritimus* belongs to another archaeal phylum, the Thaumarchaeota, which has both FtsZ and Cdv genes in the genome. Here, we used a heterologous expression system to characterize FtsZ and Cdv proteins from *N. maritimus* by investigating the ability of these proteins to form polymers. We show that one of the Cdv proteins in *N. maritimus*, the CdvB (Nmar_0816), is capable of forming stable polymers when expressed in fission yeast. The *N. maritimus* CdvB is also capable of assembling into filaments in mammalian cells. However, *N. maritimus* FtsZ does not assemble into polymers in our system. The ability of CdvB, but not FtsZ, to polymerize is consistent with a recent finding showing that several Cdv proteins, but not FtsZ, localize to the mid-cell site in the dividing *N. maritimus*. Thus, we propose that it is Cdv proteins, rather than FtsZ, that function as the cell division apparatus in *N. maritimus*.

## 1. Introduction

Cell division mechanisms in archaea, the third domain of life, have been relatively less elucidated until recent years. As opposed to eukarya and bacteria, which use actomyosin ring and FtsZ ring, respectively, for cell division, archaea appear to be more diverse in terms of their use of cell division machineries. It appears that FtsZ acts as a primary cell division apparatus in nearly all members of Euryarchaeota [1–4]. Nevertheless, FtsZ is notably absent from the other major phylum of archaea, the Crenarcheota, which consists of extremophiles that survive at extremely harsh conditions like high temperatures and high acidic environments. Recent findings strongly suggest that crenarchaeon *Sulfolobus acidocaldarius* utilizes the Cdv components (also known as endosomal sorting complex required for transport (ESCRT) in eukaryotes) for cell division [5–7]. ESCRT apparatus in eukaryotes is made up of several complexes that play important roles in different cellular processes, for instance, multivesicular body formation, membrane abscission during cytokinesis, and virus egression [8–11]. In *S. acidocaldarius*, the ESCRT-III-like CdvB (Saci_1373), Vps4-like CdvC (Saci_1372), and another gene that encodes for a coiled-coil domain protein, CdvA (Saci_1374), are arranged in an operon-like structure [5, 6]. *S. acidocaldarius* CdvB and CdcC localize to the mid cell during cell division, and their localization corresponds to the membrane ingression site between two segregated nucleoids. Overexpression of a dominant negative form of CdvC has been shown to result in enlarged cells with elevated DNA content and also cells devoid of DNA, a strong indication of cell division defects [6]. In a recent work reported by Samson et al., CdvB and CdvA were shown to cooperatively deform membranes in vitro [7], a feature that is consistent with their roles in membrane attachment, force generation, and execution of binary fission in *Sulfolobus* cells.

*N. maritimus* belongs to a phylum of archaea known as Thaumarchaeota [12, 13]. It is an ammonia-oxidizing archaeon (AOA) that contributes to the nitrification process in marine nitrogen cycle [14–16]. Interestingly, in the genome of the *N. maritimus*, there are genes that encode for both Cdv proteins and FtsZ, raising the question of which of the two components is used for cell division. A recent report by Pelve et al. showed that the *N. maritimus* Cdv proteins, but not FtsZ, localized to the mid-cell region during cell division [17], suggesting that Cdv proteins rather than FtsZ function in cytokinesis in this organism.

One of the important characteristics for cell division apparatus is the ability of one or more proteins to form polymeric structures. Actin and FtsZ have been shown to polymerize both in vivo and in vitro, and their polymerization activities are essential for cell division [18–23]. We have shown in our previous studies that tubulin-like FtsZ and actin-like MreB in bacteria form elaborate filaments in a yeast expression system [24, 25]. In this study, we seek to further understand thaumarchaeal cell division by identifying *N. maritimus* proteins that are capable of forming filament-like structures. We have focused our study on Cdv proteins and the FtsZ-like protein. We show that one of the *N. maritimus* CdvB proteins, Nmar_0816, is able to polymerize and form filament-like structures in both yeast and mammalian cells. By contrast, the FtsZ homolog in *N. maritimus*, Nmar_1262, does not polymerize or form any higher-order structure. Our findings are in agreement with the conclusions of Pelve et al., suggesting that the *N. maritimus* is likely to use Cdv proteins for cell division. 

## 2. Results and Discussion

### 2.1. Expression of *N. maritimus* CdvB and CdvC in Fission Yeast

**Figure 1:**
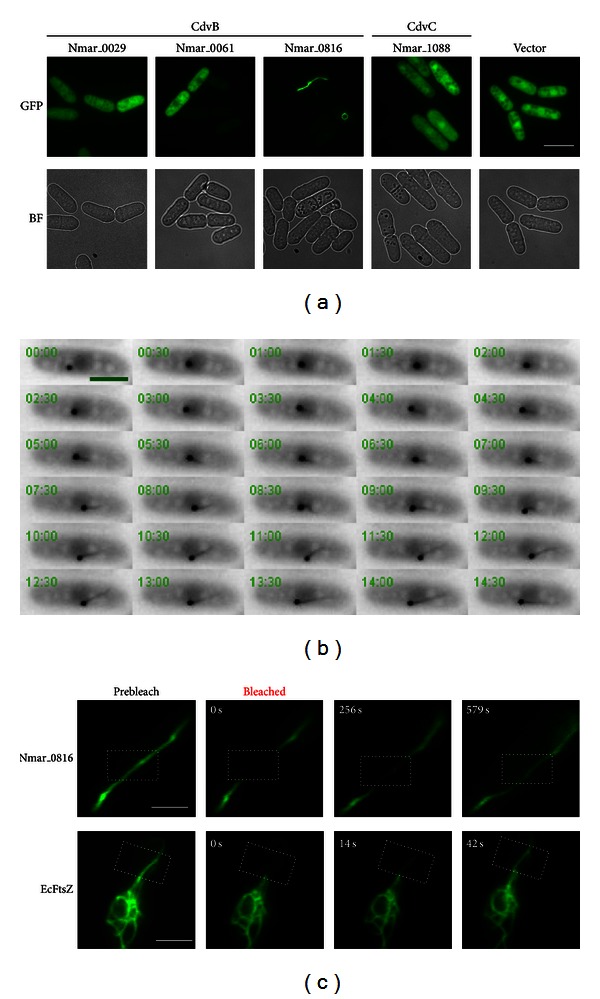
*N. maritimus* CdvB (Nmar_0816) forms filament-like structures in yeast. (a) Images of fission yeast cells expressing *N. maritimus* CdvB paralogs and CdvC fused with GFP. BF: bright field; Scale bar: 10 *μ*m. (b) Time-lapse images of Nmar_0816 polymer formation in fission yeast. Cells carrying pREP42-Nmar_0816-GFP were cultured in the absence of thiamine for 24 h at 24°C and monitored for GFP signals. Frames were captured at 15 s intervals. (Video S1). Scale bar: 5 *μ*m. (c) Time-lapse images of the Nmar_0816-GFP polymers versus the *E. coli* FtsZ-GFP polymers upon fluorescence recovery after photobleaching (FRAP). Dotted rectangle indicates bleached region. Scale bar: 3 *μ*m.

CdvB (Saci_1373) from *S. acidocaldarius* has been shown to play a central role in crenarchaeal cell division [5, 6]. In eukaryotes, ESCRT-III proteins are shown to form polymeric structures in vivo and in vitro [26–34]. In addition, several Cdv proteins from the crenarchaeon *Metallosphaera sedula* were first demonstrated to form filament-like structures in vitro in a study done by Moriscot et al. [35]. The authors showed that *M. sedula* CdvA formed helical filaments in association with DNA. Interestingly, they also demonstrated that a C-terminally deleted CdvB was capable of forming polymers even though its full-length form did not. These findings have suggested an intricate link between cell constriction/membrane deformation and the polymerizing activity of proteins involved in cell division. Since both the *N. maritimus* and the *S. acidocaldarius* CdvB proteins share substantial sequence similarity (see Figure S1 in Supplementary Material available online at http://dx.doi.org/10.1155/2013/104147), we addressed if any of the *N. maritimus* CdvB proteins could potentially polymerize into filamentous structures, an important feature that would further lend support to the claim that thaumarchaea use Cdv proteins for cell division. Since genetic manipulation techniques are yet to be developed for *N. maritimus*, we sought to answer the question using an established green fluorescence protein-(GFP-) tagging system in yeast for the examination of nonnative cytoskeletal elements. We expressed all of the three *N. maritimus* CdvB paralogs (Nmar_0029, Nmar_0061, and Nmar_0816) and the CdvC (Nmar_1088) in fission yeast with a GFP fusion at their C-terminus. Interestingly, one of the CdvB paralogs, the Nmar_0816, was found to readily form distinct polymeric structures upon expression in fission yeast (Figure 1(a)). All of the other CdvB paralogs and the CdvC examined showed only diffuse GFP signals throughout the cells, without discernible polymer formation (Figure 1(a)). It is still unclear to us why the other two CdvB paralogs (Nmar_0029 and Nmar_0061) did not form filament-like structure despite their close similarity with Nmar_0816 (Figure S1). One possibility is that fusion of GFP to the proteins might have altered the protein conformation and hence inhibited their polymerizing activity. It is also likely that Nmar_0029 and Nmar_0061 represent a distinct group of CdvB from Nmar_0816, as both Nmar_0061 and Nmar_0029 share ~50% in protein sequence identity with each other, but they share ~30% sequence identity with Nmar_0816. It is thus possible that both groups of CdvB would have distinctive properties and roles in *N. maritimus*.

Next, we took a closer look at the polymers formed by the Nmar_0816 and found that polymerized Nmar_0816 could exist in various forms ranging from simple elongated structures to closed circular and intertwined structures (Figure S2). Since these higher-order structures closely resemble those formed by cytoskeletal proteins like tubulin, actin, MreB, and FtsZ, we tested if drugs inhibiting polymerization of these cytoskeletal elements would affect the Nmar_0816 polymer formation. We found that the Nmar_0816 polymers, though sharing substantial morphological similarity to filaments formed by cytoskeletal proteins, were not affected by treatments of Latrunculin A (inhibiting actin polymerization), A22 (affecting MreB polymerization), TBZ, and MBC (inhibiting microtubule polymerization) (data not shown). These observations also established that the host cytoskeleton was dispensable for Nmar_0816 polymer formation and stability.

### 2.2. Formation of the Nmar_01816 Polymers in Fission Yeast

To understand how the Nmar_0816 polymers were formed, we took time-lapse movies of Nmar_0816-GFP in growing fission yeast cells. We found that Nmar_0816-GFP first formed an aggregate in cells with strong fluorescent signals (Figure 1(b) and Video S1). Later, elongated filament-like polymers started to emerge from the aggregate in a unidirectional manner. In cells with elaborate filaments, these polymers were also curved, circularized, and intertwined, thus forming various forms of the Nmar_0816-GFP polymers (Figure S2 and data not shown). Interestingly, in some cases, the cytokinetic apparatus of the fission yeast cells failed to cut through the Nmar_0816-GFP filaments, which resulted in extensive cytosolic vacuolization (Figure S3A and Video S2), a strong indicator of cell death in yeast (Figure S3B). Low-level expression of the Nmar_0816 did not result in polymer formation, as initial induction of Nmar_0816-GFP (first 16 hours) only resulted in cells with fluorescent signal but no filament-like structures. A further 4–6 hours induction was needed for the polymer formation (data not shown). Our experiments suggest that Nmar_0816 protein has to reach a likely threshold level before aggregation and polymerization can be initiated. 

Next, we sought to understand the protein turnover property of the Nmar_0816 polymers by fluorescence recovery after photobleaching (FRAP). FRAP analysis revealed a very slow signal recovery for Nmar_0816 polymers upon photobleaching as compared to the *E. coli* FtsZ (~2% signal recovery for Nmar_0816 polymers at 56 seconds postbleaching as compared to ~37% signal recovery for FtsZ filaments at 52 seconds postbleaching), indicating that the higher-order structures formed by the Nmar_0816 protein were stable (Figure 1(c)). Collectively, our data suggest that the Nmar_0816 is capable of forming stable higher-order polymers in fission yeast cells. In contrast to rapid turnover of the FtsZ protein that ensures proper control of *E. coli* division, a stable CdvB might be needed in *N. maritimus* for division. It is also likely that the *N. maritimus* CdvB requires other factors like CdvC to regulate its turnover.

### 2.3. The *N. maritimus* CdvB (Nmar_0816) Polymerizes into Higher-Order Structures in Mammalian NRK Cells

**Figure 2:**
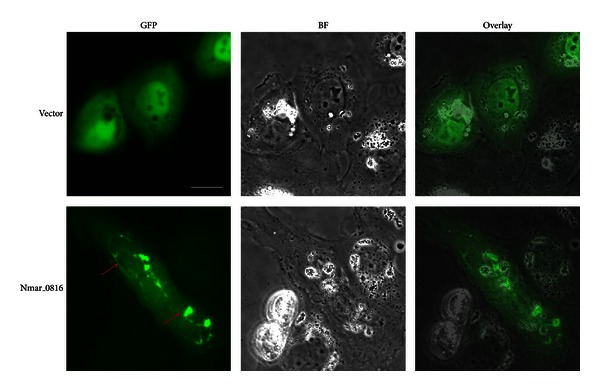
Nmar_0816 forms filament-like structures in mammalian cells. Images of mammalian NRK cells expressing GFP-Nmar_0816. Red arrows point to filament-like structures. BF: bright field; Scale bar: 7 *μ*m.

To determine if the formation of the Nmar_0816 polymers was limited to the cellular context of fission yeast, we transiently transfected mammalian NRK cells with a construct expressing the Nmar_0816 with a GFP fusion at its N-terminus. The transfected cells with GFP-Nmar_0816 showed filament-like polymers (Figure 2). By contrast, in the control cells transfected with GFP-containing vector, only diffuse GFP signals were seen. Intriguingly, in some transfected cells, the GFP-Nmar_0816 seemed to localize to the rim of enlarged endosomes (Figure S4), a feature similar to that observed upon overexpression of truncated hVps2-1, hVps24, and hSnf7-1 in mammalian cells [36]. Vps20, a component of ESCRT-III, contains a myristoylation site that potentially facilitates its direct association with the membrane. It has been shown that Vps20 mediates the recruitment of Vps24 and Vps2 to the membrane [8]. Interestingly, Nmar_0816 lacks the putative lipid modification site, and it shares 12% and 5% sequence identity with mammalian Vps24 (hVps24) and hVps20, respectively (Figure S5). As ESCRT-III members have been shown to interact with each other, it is possible that Nmar_0816 is targeted to the rim of endosomes through its potential interaction with the mammalian Vps20. Nevertheless, further experiments are needed to demonstrate if the targeting of CdvB to the rim of endosomes is specific. Our findings suggest an interesting link between the *N. maritimus* CdvB and the mammalian ESCRT-IIIs. Nevertheless, we have not observed endosome association of the Nmar_0816-GFP in yeast. Different cellular factors may have contributed to distinct forms and localization of the Nmar_0816 in fission yeast and in mammalian cells. Taken together, our data showed that the Nmar_0816 was able to assemble into polymeric structures not only in fission yeast, but also in mammalian cells. 

### 2.4. Evaluation of the *N. maritimus *CdvB (Nmar_0816) Filament-Forming Property

**Figure 3:**
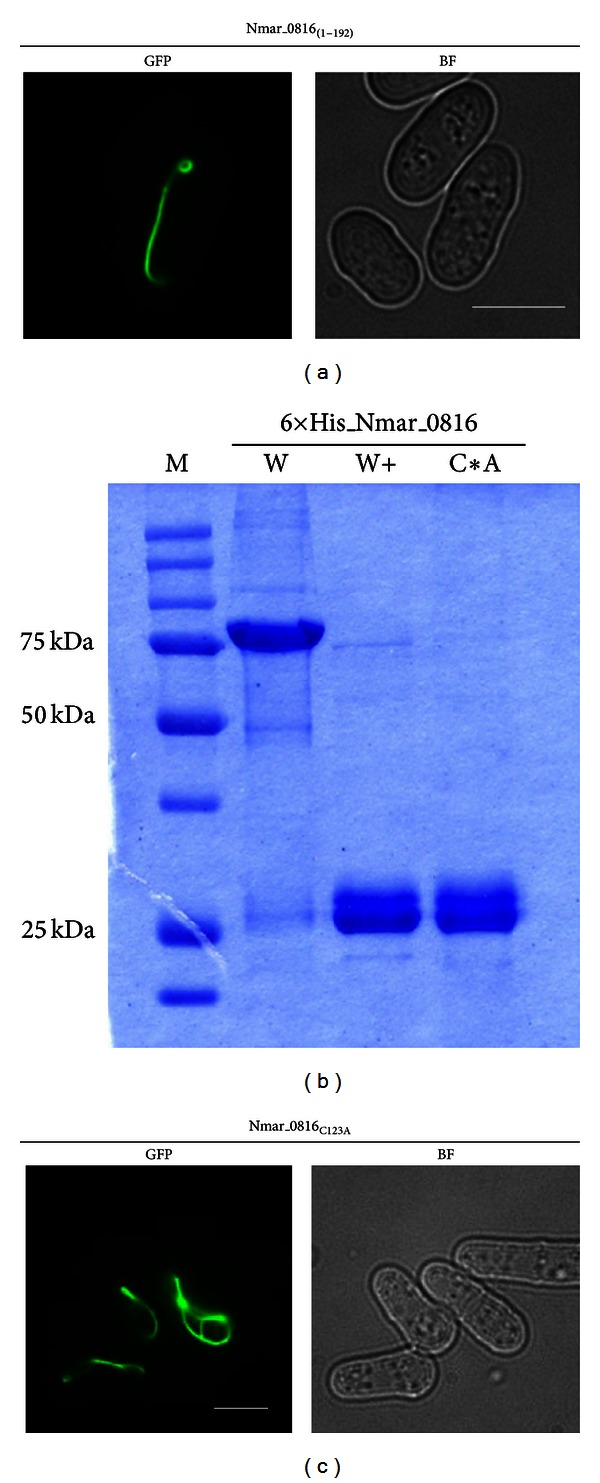
The core domain of the CdvB is necessary for Nmar_0816 polymer formation in yeast. (a) Images of yeast cells expressing Nmar_0816_(1–192)_-GFP. Scale bar: 7 *μ*m. (b) Expression and purification of Nmar_0816 in *E. coli*. W: 6 × His-Nmar_0816, nonreducing condition. W+: 6 × His-Nmar_0816, in the presence of 5%  *β*-mecaptoethanol. C*A: 6 × His-Nmar_0186_C123A_, nonreducing condition. (c) Images of yeast cells expressing Nmar_0186_C123A_-GFP. Scale bar: 5 *μ*m.

Previous studies have shown that the core domain of the eukaryotic ESCRT-III was essential and sufficient for polymerization [33, 36]. We sought to understand if this filament-forming property was also conserved in the *N. maritimus* CdvB. We generated N-terminal (1–108 aa) and C-terminal (109–206 aa) Nmar_0816-GFP, in which the core domain was disrupted in both cases. Not surprisingly, expression of both GFP-fusion proteins resulted in diffuse GFP signals without discernible polymeric structure (data not shown). By contrast, a small C-terminal deletion of the Nmar_0816 that retained its core domain (1–192 aa) formed elaborate filaments similar to the full length Nmar_0816 (Figure 3(a)). As the C-terminal deletion also removed the putative MIT-interacting motif 2 (MIM2, for the interaction with MIT domain of the CdvC protein) of the Nmar_0816, it is also likely that the formation and stability of the Nmar_0816 polymers are independent of its interaction with the CdvC protein. Since the putative MIM2 motif in Nmar_0816 is not highly conserved with those of Saci_1373 and mammalian CHMP6 (Figures S1 and S5), it is not clear if it is capable of interacting with CdvC. Taken together, we showed that the core domain of the CdvB was sufficient for Nmar_0816 polymerization (Table 1 and Figure 3(a)). Interestingly, it is still not known to us why the other *N. maritimus* CdvB paralogs (the Nmar_0029 and the Nmar_0061) did not polymerize as both of them possess similar core domains for such a function. It is possible that interference from the GFP fusion might have negative impact on the polymerizing activity of the other two CdvB paralogs. In addition, sequence variation in the C-terminal regions (Figure S1) might have contributed to distinctive regulation of the CdvB paralogs for polymerization. It is also possible that specific and individualistic differences of protein folding may have contributed to such distinction. 

To further investigate the protein properties of the Nmar_0816, we expressed recombinant Nmar_0816 in *E. coli* and found that purified Nmar_0816 existed as both monomer and dimer/multimer under nonreducing condition (Figure 3(b)). The dimer/multimer forms were readily resolved into monomeric form of ~25 kDa in the presence of reducing agents. To examine if the dimeric/multimeric forms of the Nmar_0816 were due to the presence of a disulfide bond, we mutated the codon coding for cysteine into alanine and found that the mutated Nmar_0816 was resolved only as a monomer in nonreducing SDS-PAGE. As polymerization could be initiated from monomeric or dimeric/multimeric forms of a protein, we were interested to know if the dimeric/multimeric forms of the CdvB protein served as protein nucleators for the polymerization. To address this issue, we expressed the mutated form of the Nmar_0816 in yeast to examine if its polymerization activity would be disrupted if there is only the monomeric form present. As shown in Figure 3(c), the cysteine to alanine mutated Nmar_0816 effectively polymerized into filament-like structures that were indistinguishable from the wild type suggesting that dimeric/multimeric forms of Nmar_0816 are not required for its polymerization. It is noteworthy that the Nmar_0816 is the only CdvB paralog that contains a cysteine residue (Figure S1, C123). Even though we have ruled out that polymerization requires dimeric/multimeric forms of the Nmar_0816, it will be interesting to know if the ability of the Nmar_0816 to dimerize/multimerize has any significant role in its protein folding and stability, or function in vivo. 

### 2.5. The *N. maritimus* FtsZ Is Substantially Different from the *E. coli* FtsZ and Does Not Polymerize into Filament

**Figure 4:**
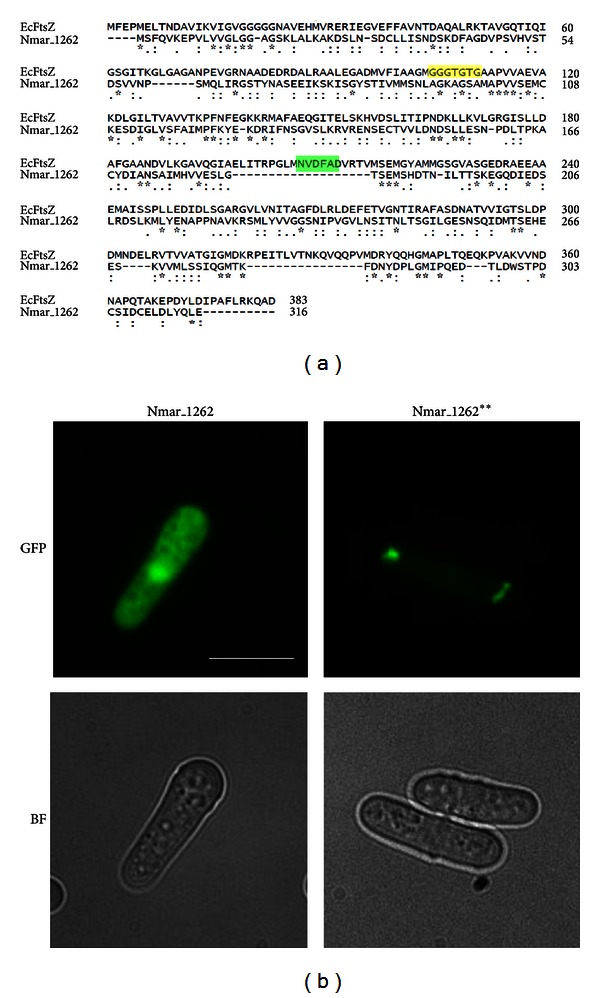
*N. maritimus* FtsZ-GFP (Nmar_1262) does not form filament-like structures in yeast. (a) Sequence alignment of the Nmar_1262 with the *E. coli* FtsZ (EcFtsZ). Regions highlighted in yellow and green in the EcFtsZ sequence are two conserved motifs for tubulin/FtsZ, GGGTGTG, and NVDFAD (T7-loop) for GTP binding and hydrolysis, respectively. Sequence alignment was performed using ClustalW2 program available from the website http://www.ebi.ac.uk/Tools/msa/clustalw2/. (b) Images of yeast cells expressing Nmar_1262-GFP and Nmar_1262**-GFP, in which the presumptive GTP-binding motif AGKAGSA was replaced with the conventional GGGTGTG. Scale bar: 10 *μ*m.

*N. maritimus* genome contains a gene for FtsZ. Since FtsZ is used in bacteria and euryarchaea as the cell division apparatus and is capable of polymerization, we asked if the *N. maritimus *FtsZ (Nmar_1262) has similar polymerization activity. We aligned and compared the sequence of the *Nitrosopumilus* FtsZ with the *E. coli* FtsZ. As shown in the sequence alignment, the *N. maritimus* FtsZ does not share substantial sequence similarity with the *E. coli* FtsZ even at the most conserved domains for GTP binding and hydrolysis (T7-loop) (Figure 4(a), highlighted in yellow and green, resp.). We have previously shown that expression of the *E. coli* FtsZ in yeast resulted in elaborate FtsZ filaments [25]. However, the *N. maritimus* FtsZ did not show any polymeric structure when expressed in yeast (Figure 4(b)). Since the *N. maritimus* FtsZ does not possess a conserved motif for GTP binding [37], we replaced the GTP-binding motif of *N. maritimus* FtsZ with the conserved GTP-binding motif (GGGTGTG) of *E. coli* FtsZ. The mutant *Nitrosopumilus* FtsZ (Nmar_1262**) formed small aggregate-like structures in yeast but still did not polymerize into filaments (Figure 4(b)). Interestingly, *E. coli* FtsZ carrying a mutation in the T7 hydrolysis loop (D209A, Figure 4(a)) formed similar aggregate-like structures when expressed in fission yeast (Figure S6). Mutations in the T7 loop are known to disrupt FtsZ polymerization but not GTP binding [38, 39]. *N. maritimus* FtsZ seems to lack the conserved T7 loop (NVDFAD, Figure 4(a)). It is likely that the altered localization and morphology of fluorescent foci in Nmar_1262** might be due to GTP binding in the absence of an active T7 loop. Collectively, our result suggests that the *N. maritimus* FtsZ might have undergone multiple nucleotide changes following loss of GTP-binding activity such that even replacement of GTP-binding motif is not sufficient to restore its ability to polymerize into filaments.

Our findings and those from the study of Pelve et al. have both suggested that *N. maritimus* is likely to use Cdv proteins for cell division even though there is also a gene encoding for FtsZ-like protein. This raises another intriguing question on what is the function of the FtsZ in *N. maritimus* if not for cell division. However, from the sequence alignment with *E. coli* FtsZ, it is clear to us that *N. maritimus* FtsZ does not have a conserved GTP-binding motif (hence, might not bind to GTP) and is completely lacking the T7-loop for GTP hydrolysis. Thus, it is likely that the *N. maritimus* FtsZ has lost its cell division function. Interestingly, Thaumarchaeota has been suggested to have diverged before the speciation of Euryarchaeota and Crenarchaeota [12]. It is not impossible that an ancestral lineage of archaea with both FtsZ-like and Cdv proteins had evolved to give rise to two distinct archaeal lineages where one uses Cdv proteins for cell division and loses the FtsZ, while the other relies on FtsZ and does away with Cdv proteins. In that way, thaumarchaea might represent a “living fossil” in which Cdv proteins are being used for cell division, while FtsZ is losing its functional role in the evolution of cell division machinery.

An interesting aspect of CdvB is that it often exists as multiple paralogs in the genomes (three in *N. maritimus* and four in *S. acidocaldarius*). It would be intriguing to know if these paralogs have overlapping functions. In *S. acidocaldarius*, only one of the CdvB proteins (Saci_1373) has been shown to be involved in cell division [5, 6]. Two other CdvB paralogs (Saci_0451 and Saci_1416) have been suggested to have originated from a more recent gene duplication event [40]. Interestingly, these two paralogs were found to be in secreted membrane vesicles [41], indicating that they might play a distinct role from Saci_1373 in *S. acidocaldarius*. In *N. maritimus*, based on the sequence identities, it is likely that the polymer-forming Nmar_0816 and the other two paralogs (Nmar_0029 and Nmar_0061) form distinct groups of CdvB that would have played different roles. Nevertheless, more experiments need to be done to reveal their respective functions in *N. maritimus*. It would also be interesting to understand which of the CdvB paralogs are essential and which are not for the organisms. Gene deletion analysis would provide a quick glimpse on this point. 

In summary, we have shown that one of the *N. maritimus* CdvB paralogs, Nmar_0816, was capable of assembling into filaments. By contrast, the FtsZ did not polymerize in our assay. As cytoskeletal polymers are involved in cell division, we conclude that *N. maritimus* likely uses CdvB as its cell division machinery.

## 3. Experimental Procedures 

### 3.1. Plasmid Preparation

Coding sequences for the *N. maritimus* Cdv components (Nmar_0029, Nmar_0061, Nmar_0816, and Nmar_1088) and the FtsZ (Nmar_1262) were codon optimized, synthesized, and cloned into pUC57 plasmids by commercial gene synthesis service (GenScript Inc. USA). *S. pombe* GFP-tagging expression vector pREP42-GFP containing a uracil biosynthesis gene for auxotroph selection was from our lab collection. Expression of the GFP fusion proteins with pREP42-GFP vector was under the control of a mid-strength thiamine-repressible nmt promoter [42].

### 3.2. Gap-Repair Cloning and Yeast Transformation

Gap-repair cloning was performed as described [43]. Gene-specific fragments (see Table S1 for the primer list) from *N. maritimus* with ~50nts overlapping regions from pREP42-GFP at N- and C-terminus were obtained by PCR amplification using high fidelity taq polymerase (Roche) according to manufacturer's instruction. Different pUC57 plasmids carrying codon-optimized *N. maritimus* genes were used as templates. PCR fragments were analyzed using agarose gel electrophoresis. The pREP42-GFP vector was linearized by *BamHI* and *NdeI* double digestion (New England Biolabs) and purified using column purification kit (QIAGEN). PCR fragments and linearized pREP42-GFP were mixed in 10 : 1 ratio and transformed into MBY192 strain (*ura4*-D18, *leu1*-32, h-) using lithium acetate method as previously described [44]. Briefly, yeast cells were grown to an optical density of 0.5. Cells were rinsed once with sterile water and washed once with 1x LiAc/TE solution (100 mM lithium acetate, 10 mM Tris-HCl, 1 mM EDTA, pH 7.5). Cells were resuspended in 100 *μ*L of 1x LiAc/TE solution and incubated with respective plasmids for 10 min at room temperature. The cells were further incubated with 240 *μ*L of PEG/LiAc/TE solution (LiAc/TE solution with 40% polyethylene glycol 4000) for 30 min at 30°C. 42 *μ*L of dimethyl sulfoxide (DMSO) was added to the cells prior to heat shock at 42°C for 5 min. After heat shock, cells were washed once with sterile water and plated on Edinburgh minimal medium (EMM) supplemented with amino acids and 15 *μ*M of thiamine. After 4-5 days of growth, at least 20 colonies from each of the transformants were picked and re-streaked on fresh supplemented EMM plates in the absence of thiamine and incubated at 30°C for 4-5 days. Colonies were then examined for fluorescence under fluorescence microscope. Transformants with GFP signals were grown in liquid cultures for detailed examination. To further verify filament-forming Nmar_0816-GFP, cells were transformed with pREP42-Nmar_0816-GFP plasmid constructed by conventional restriction-based cloning method. The transformants of pREP42-Nmar_0816-GFP showed filament-forming properties that were indistinguishable from transformants obtained by gap-repair cloning. To construct pREP42-Nmar_1262**-GFP (replacement of variant GTP binding motif AGKAGSA with conventional GGGTGTG), two independent N-terminal and C-terminal fragments were obtained by PCR amplification using primers incorporating the corresponding changes of nucleotide sequences (see Table S1 for primer sequences). Both fragments were used for an overlapping PCR to get final PCR fragment of Nmar_1262 with a swap of GTP binding motif (Nmar_1262**). The PCR fragment of Nmar_1262** was then used for subsequent gap repair cloning.

### 3.3. Cell Culture, Transfection, and Expression of GFP-Nmar_0816 in NRK Cells

For the expression of GFP-Nmar_0816 in mammalian cells, full length Nmar_0816 was amplified and cloned into pEGFP-C1 vector (Clontech). NRK cells were maintained in Kaighn's modified F12 (F12K, Sigma) medium supplemented with 10% fetal bovine serum (FBS), 1 mM L-glutamine, 100 U/mL penicillin, and 100 *μ*g/mL streptomycin (GIBCO) at 37°C and 5% CO_2_. For transfection, cells were grown on a cover slip chamber to 60%–70% confluency. Cells were rinsed once with Opti-MEM I medium (LifeTechnologies) immediately before transfection. The rinsed cells were then transfected with 1 *μ*g each of the pEGFP-C1-Nmar_0816 and the pEGFP-C1 using Lipofectamine 2000 reagent (Invitrogen) according to manufacturer's instructions. After 4 h of incubation, the medium containing DNA-Lipofectamine was replaced with the F12K medium containing 10% FBS, and the cells were cultured for an additional 14–16 h before imaging.

### 3.4. Protein Expression and SDS-PAGE Analysis

Full length Nmar_0816 was PCR amplified (see Table S1 for primers information) and cloned into pQE30 vector (QIAGEN) for expression as 6 × His-Nmar_0816 recombinant protein in *E. coli* M15. The recombinant 6 × His-Nmar_0816 was induced with 1 mM IPTG and purified on nickel column (QIAGEN) according to manufacturer's instruction. To express the 6 × His-Nmar_0816_C123A_ (cysteine to alanine mutation) in *E. coli*, two independent N-terminal and C-terminal PCR fragments were obtained using primers incorporating the cysteine to alanine mutation in nucleotide sequences (see Table S1 for primer sequences). Overlapping PCR was performed using both N- and C-terminal PCR fragments to get a final PCR fragment of the Nmar_0816 with cysteine to alanine mutation. The Nmar_0816_C123A_ fragment was then cloned into pQE30 vector for expression and purification. The purified recombinant proteins were analyzed by 10% SDS-PAGE.

### 3.5. Microscopy and FRAP Analysis

For the epifluorescence imaging of yeast, images were acquired on an Olympus IX71 inverted microscope equipped with a charge-coupled device (CCD) camera (CoolSNAP ES, Photometrics), a 100x/1.45 NA Plan Apo objective lens (Olympus), and Metamorph (v.7.6) software. Fluorescence recovery after photobleaching (FRAP) was performed using Zeiss Meta 510 inverted confocal microscope with a 100x/1.25 NA Apochromat objective lens. For fluorescence imaging in mammalian NRK cells, the images were taken using Axiovert 200 M inverted microscope (Carl Zeiss) with a 100x/1.30 NA Plan-Neofluar lens or Zeiss LSM Meta 510 inverted confocal microscope with a 100x/1.4 NA Plan-Apochromat lens. All images from Axiovert 200 M were acquired using a cooled CCD camera (CoolSNAP_HQ_, Roper Scientific) and MetaView imaging software (Universal Imaging). Images were processed with ImageJ software (http://rsb.info.nih.gov/ij/) for presentation.

## Supplementary Material

The Supplemental information includes 2 videos (formation of Nmar_0816-GFP filaments in yeast (Video S1); yeast cell death caused by failure to dissolve Nmar_0816 polymers during cell division (Video S2)), 6 figures (multiple sequence alignment of the CdvB genes from *N. maritimus* and *S. acidocaldarius* (Figure S1); diverse polymeric structures formed by Nmar_0816 in yeast (Figure S2); yeast cell death caused by failure to dissolve Nmar_0816 polymers (Figure S3); localization of Nmar_0816 to the rim of endosomes in NRK cells (Figure S4); multiple sequence alignment of the CdvB genes from *N. maritimus* and ESCRT-III genes from human and fission yeast (Figure S5); expression of *E. coli* FtsZ mutant (D209A) in yeast (Figure S6)), and 2 tables (list of primers used in this study (Table S1); list of yeast strains used in this study (Table S2)).

## Figures and Tables

**Table 1:** Summary on the domain analysis of the Nmar_0816 (1–216 aa) protein for its polymerization activity in yeast.

	Polymerization activity
Nmar_0816_(1–216)_	Yes
Nmar_0816_(1–108)_	No
Nmar_0816_(109–216)_	No
Nmar_0816_(1–192)_	Yes
Nmar_0816_C123A_	Yes

In Nmar_0816_(1–108)_ and Nmar_0816_(109–216)_, the core domain (1–181 aa) was disrupted. In Nmar_0816_C123A_, the cysteine residue was mutated to alanine. In Nmar_0816_(1–192)_, the putative MIM2 motif was removed.
