# Supplementary material for: The Nitrosopumilus maritimus CdvB, but Not FtsZ, Assembles into Polymers
Source: Archaea. 2013 Jun 2;2013:104147. doi: 10.1155/2013/104147 (PMC3684127; doi:10.1155/2013/104147)
Supplement: Supplementary file 1 — The Supplemental information includes 2 videos (formation of Nmar_0816-GFP filaments in yeast (Video S1); yeast cell death caused by failure to dissolve Nmar_0816 polymers during cell division (Video S2)), 6 figures (multiple sequence alignment of the CdvB genes from N. maritimus and S. acidocaldarius (Figure S1); diverse polymeric structures formed by Nmar_0816 in yeast (Figure S2); yeast cell death caused by failure to dissolve Nmar_0816 polymers (Figure S3); localization of Nmar_0816 to the rim of endosomes in NRK cells (Figure S4); multiple sequence alignment of the CdvB genes from N. maritimus and ESCRT-III genes from human and fission yeast (Figure S5); expression of E. coli FtsZ mutant (D209A) in yeast (Figure S6)), and 2 tables (list of primers used in this study (Table S1); list of yeast strains used in this study (Table S2)). [file 104147.f1.zip › Supplementary figures.pdf]

Figure S1

```
Saci_1416       MADVNDFLRNWGGRQEPTISEKIKNLFKSQQPLRYRLVMANYRLRTTISRLDVYISKLQE 60
Saci_0451       MTAIYILTMISKEFQKIWEGNQKVKIPKSKDPLKYRLVQAQYRVRSMVSRLDVYIGRMQE 60
Nmar_0061       ---MPNFDKTWARQETQSVTGKLREAVKPQGALKPRIQTAVNKLQVQISKMDSMLGKLHE 57
Nmar_0029       ---MANLSNKWTKPPTTSITEKIGDTVKPKGALKPRVQEGVKRLRLQIQKLDKMLSGLKE 57
Nmar_0816       -----MLSNSWNNTGGESISQKVMGKVKPDEPLKNKIDFAQKKLQFQITKLEGINEKLRV 55
Saci_1373       ---------MFDKLSIIFNSDR-----KRKVHLSKAITEISLKLKEQQDRLDEAIRRLRE 46
Saci_1601       -------------------------------MKKRTIAELLTDIRMAKYKIDMWISKAEN 29
                                                    :      ::    :::      .

Saci_1416       RDRSLFEKVVESQISKDSARAAMYANEIAEIRKITKQLLTTEIALEQVQLRLETITEIGD 120
Saci_0451       RDKVLFERVVESQMSKDQSRAAMYANELAEIRKITKQLLTTQIALEQVSLRLETVTELGD 120
Nmar_0061       RDAQLFQRVVTAMQQHDTSTSRVLSNELAEIRKVTKMLGNARMSLEQVQLRLTTIHDLGD 117
Nmar_0029       RDEQLFARIVEATQKHDTQTSKVLGNELAEIRKVIKIMSSARVALEQIELRLTTFSDLGD 117
Nmar_0816       KHDQIFEKIVSAHKSNKNAYAQAYANELAQVRKMKNMVSGAKLSMEQVKLRLDTVSELGD 115
Saci_1373       RDKDLFEKVIRSQIEGDIARATIYAQEISDIRKMIKIIYTAYLAIEKVRLKLDTVQELQG 106
Saci_1601       RNK-ALERLSLSNIGRFPLLSKEYIKETELTRKYIVTLVQLKILLEILEIRLETLIILGN 88
                :.   : ::  :        :    :*    **    :    : :* : ::* *.  : .

Saci_1416       IFTSLVPVIGVIRELRNVMKGVMPELSIELADLEEGLQEVVLEA-GEFTGARVDFATSSP 179
Saci_0451       IFANLIPVMGVINELKTSLKGVMPELSIELGELGEGLQEIVIEA-GEFSGVSGISATYSP 179
Nmar_0061       AMVAIGPAMSTMKGLKSSLGRFMPEADSELNTMTQTLNGLMMDS-LAGDSFNMESDVSSE 176
Nmar_0029       TVVAIMPTMGLMKNLKSSLGKVMPGAEAEIGQMAEMLGGFMTES-FSGDAAFGMDETTNA 176
Nmar_0816       VVVTLSPCMSIIKGLAPSLNGIMPEANASMQDLSSVLGDVMSGSSVDLGDSMSVGPETNA 175
Saci_1373       VSLVLFPVMRILGELKEQVRGIAPEVALALDSITSSVNSIAIET-GALSEKTFVPTVADE 165
Saci_1601       VVTYLSPLVEALNELKGQLG-ASIEFSPIIDEIIETIRTVYIAP---NTVQQSPQINVKE 144
                    : * :  :  *   :          :  : . :  .   .              .

Saci_1416       EARKILDEASAVAEQRMKEKFPSLPSFATSVDQKTNANQK-------------------- 219
Saci_0451       EARQILEEASVVAEQRMKEKFPSLPAAGITQQQKS------------------------- 214
Nmar_0061       ETEKILQEASAVAEQQIGDKFPSVPSSTGLSSGTSTSTFE-------------------- 216
Nmar_0029       ESDAILKEAAAVAESSAGQMFPSVPTESAAST-ESTSKFY-------------------- 215
Nmar_0816       DTLAILEEAQGVIAGQTKASIPDVPDSLKQQIVEKKTDIFI------------------- 216
Saci_1373       QAKQIMEEAQKMAEVKVRELLPELPHPPSELPKRVAKQVQSSNKKSLSEDMILNYIKTTG 225
Saci_1601       EARQLLKEAEDVAKKELKENYKIEI----------------------------------- 169
                ::  ::.**  :

Saci_1416       ------------------------------------
Saci_0451       ------------------------------------
Nmar_0061       ------------------------------------
Nmar_0029       ------------------------------------
Nmar_0816       ------------------------------------
Saci_1373       GFIDVDYIAKNFDVSKDEVFNVLRRLEEKGLIVLEG 261
Saci_1601       ------------------------------------
```

Figure S1: Multiple sequence alignment of the CdvB genes from *N. maritimus* and *S. acidocaldarius*.
Highly conserved and partially conserved residues are highlighted as asterisks and dots, respectively. CdvA binding site and MIM2 motif in Saci_1373 [7] are highlighted in red and purple, respectively. Putative MIM2 motif in Nmar_0816 is highlighted in blue (Figure S5). Cysteine residue in Nmar_0816 is highlighted in green. The multiple sequence alignment was performed using ClustalW2 program.

Figure S2

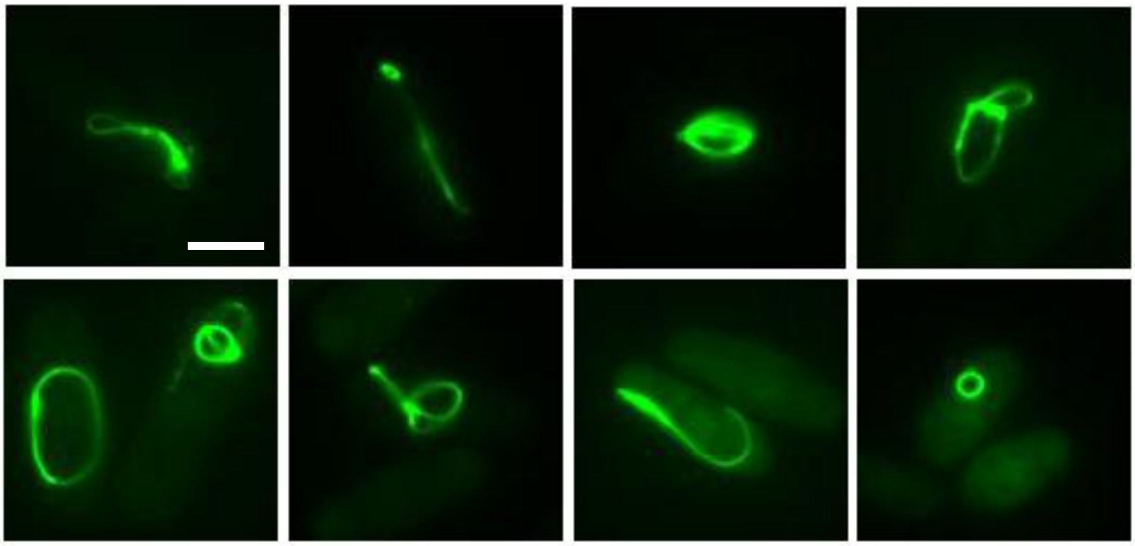

Figure S2: Nmar_0816 forms diverse polymeric structures in yeast
Images of yeast cells expressing Nmar_0816-GFP. Scale bar: 5 µm.

Figure S3

A

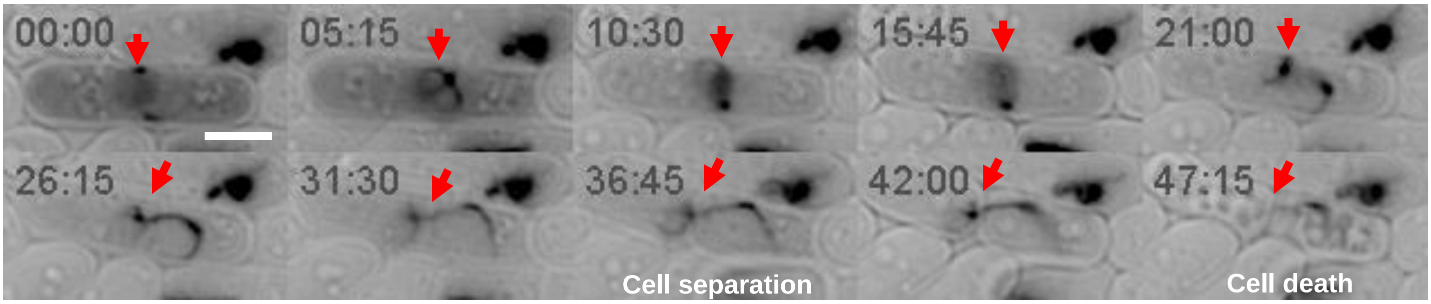

00:00
05:15
10:30
15:45
21:00
26:15
31:30
36:45
42:00
47:15
Cell separation
Cell death

B

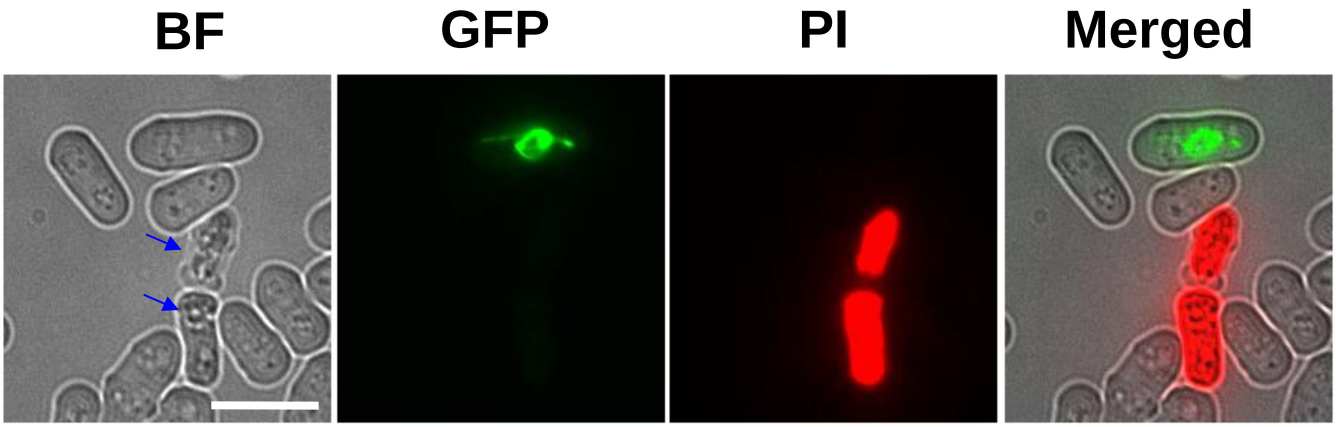

BF
GFP
PI
Merged

Figure S3: Failure to dissolve Nmar_0816 polymers during yeast cell division causes cell death.
(A) Time-lapse images of yeast cells expressing Nmar_0816-GFP polymers during cell division. Red arrows indicate mid-cell regions. Extensive cytosolic vacuolization of cells at the end of the cell division indicates cell death. Video S2. Scale bar: 5 μm. (B) Images of yeast cells expressing Nmar_0816-GFP and stained with 10 μg/mL propidium iodide to reveal cell death. Blue arrows point to extensively vacuolated cells. BF: bright field; GFP: Nmar_0816-GFP; PI: propidium iodide staining. Scale bar: 10 μm.

Figure S4

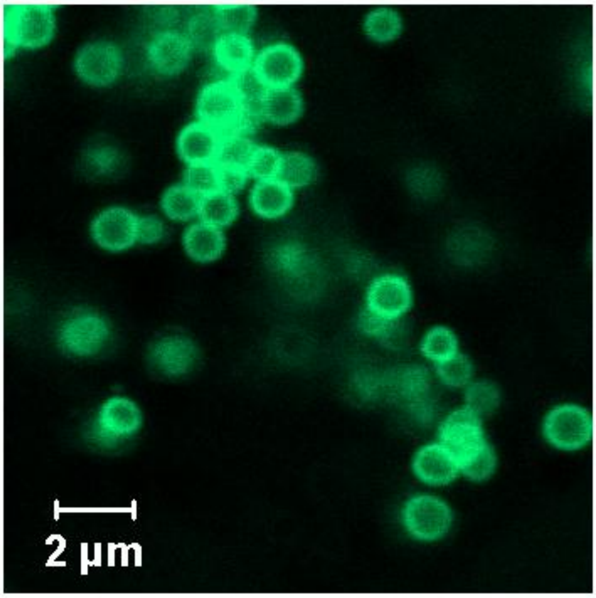

Figure S4: Nmar_0816 localizes to the rim of endosomes in NRK cells.
Image of NRK cells expressing GFP-Nmar_0816.

Figure S5

```
hVps20          ---MGNLFGRKKQSRVTEQDKAILQLKQQRDKLRQYQKRIAQQLERERALARQLLRDGRK 57
SpVps20         ---MG-----VNSSKINDKDRSILSIKEQRDKLLRYSKRLEKIEQLEIDIARKCLRDSDK 52
hSnf7-1         MSGLGRLFGKGKKEKGPTPEEAIQKLKETEKILIKKQEFLEQKIQQELQTAKKYGTK-NK 59
SpSnf7          MSGFLRWFG-GNRSKD-TTKDTIVRFQEMLALYDKKEEVLERQIAEQTEIARKNATT-NK 57
Nmar_0061       ----------MPNFDKTWARQETQSVTGKLREAVKPQGALKPRIQTAVNKLQVQISKMDS 50
Nmar_0029       ----------MANLSNKWTKPPTTSITEKIGDTVKPKGALKPRVQEGVKRLRLQIQKLDK 50
Nmar_0816       ------------MLSNSWNNTGGESISQKVMGKVKPDEPLKNKIDFAQKKLQFQITKLEG 48
hVps24          ---MGLFGKTQEKPPKELVNEWSLKIRKEMRVVDRQIRDIQREEEKVKRSVKDAAKKGQK 57
SpVps24         --MQTVRSYFFGPTPQEQNRKWQSIIRKEQRQLDRQVYHLKAGRKKAEVQLKQLAKQSDI 58
                                         .        :    :           :        

hVps20          ERAKLLLKKKRYQEQLLDRTENQISSLEAMVQSIEFTQIEMKVMEGLQFGNECLNKMHQV 117
SpVps20         RGALRALKAKKLYSGLITQTYGQLGNIEQLLSTIEFTLIQKDVMFGLQEGTNLIRQLQAD 112
hSnf7-1         RAALQALRRKKRFEQQLAQTDGTLSTLEFQREAIENATTNAEVLRTMELAAQSMKKAYQD 119
SpSnf7          RLALTALKRKKMHENELVKIEGSRNNIEQQLFSIQNANLNFETLQAMRQGAEAMKSIQRG 117
Nmar_0061       MLGKLHERDAQLFQRVVTAMQQH-DTSTSRVLSNELAEIRKVTKMLGNARMSLEQVQLRL 109
Nmar_0029       MLSGLKERDEQLFARIVEATQKH-DTQTSKVLGNELAEIRKVIKIMSSARVALEQIELRL 109
Nmar_0816       INEKLRVKHDQIFEKIVSAHKSN-KNAYAQAYANELAQVRKMKNMVSGAKLSMEQVKLRL 107
hVps24          DVCIVLAKEMIRSRKAVSKLYASKAHMNSVLMGMKNQLAVLRVAGSLQKSTEVMKAMQSL 117
SpVps24         TNMRILAKEIARANRHGKRLAESKALLGSLSLQLNDQMAMLKIQGTMQSSTKIMQDVSSL 118
                       :                          :                   .     

hVps20          MSIEEVERILDETQEAVEYQRQIDELLAGSFTQ---EDEDAILEELSAITQEQ------- 167
SpVps20         MPLERVGRICNDRDEAMSYVDEVNDMLQGRMSR---DQEDEIQEELDSLIREQEDEKVKD 169
hSnf7-1         MDIDKVDELMTDITEQQEVAQQISDAISRPMGFGDDVDEDELLEELEELEQEE------- 172
SpSnf7          MDADKVDQIMDKIRDQQTISEEISTMISTPVGLNAEIDEDELANELDELQQME------- 170
Nmar_0061       TTIHDLGDAMVAIGPAMSTMKGLKSSLGRFMPEAD-SELNTMTQTLNGLMMDS------- 161
Nmar_0029       TTFSDLGDTVVAIMPTMGLMKNLKSSLGKVMPGAE-AEIGQMAEMLGGFMTES------- 161
Nmar_0816       DTVSELGDVVVTLSPCMSIIKGLAPSLNGIMPEAN-ASMQDLSSVLGDVMSGSS------ 160
hVps24          VKIPEIQATMRELSKEMMKAGIIEEMLEDTFESMD--DQEEMEEEAEMEIDR-------- 167
SpVps24         IRLPQLSETMRNLSMELTKAGVLEEMRDEMFLPVE--DDEELMDLADEDEEVQEILTKYN 176
                     :                :       .      .   : .                

hVps20          IELP---------EVPSEPLPEKIP---ENVPVKARPRQAELVAAS----------- 201
SpVps20         LEKPGFTPSTGVDVLPSVPLKNAIPSLDESFPKAASVSNTSSAVVIDEELRKDPVLG 226
hSnf7-1         --LAQELLNVGDKEEEPSVKLPSVPS--THLPAGPAP-KVDEDEEALKQLAEWVS-- 222
SpSnf7          --LDSKMLGA-EKPPVHTPAVPAVPSQVKDLPSISKPQELDEEEELRKLQAEFSL-- 222
Nmar_0061       LAGDSFNMESDVSSEETEKILQEASAVAEQQIGDKFPSVPSSTGLSSGTSTSTFE-- 216
Nmar_0029       FSGDAAFGMDETTNAESDAILKEAAAVAESSAGQMFPSVPTESAAST-ESTSKFY-- 215
Nmar_0816       VDLGDSMSVGPETNADTLAILEEAQGVIAGQTKASIPDVPDSLKQQIVEKKTDIFI- 216
hVps24          ILFEITAGALGKAPSKVTDALPEPEPPGAMAASEDEEEEEEALEAMQSRLATLRS-- 222
SpVps24         VIPAPSEKAADAATHREQSLKQALPSLSNGIAKDSTEIDEEQLLDIRDKLDALKS-- 231
```

Figure S5: Multiple sequence alignment of the CdvB genes from *N. maritimus* and ESCRT-III genes from human and fission yeast.
Partially conserved residues are highlighted as dots. hSnf7-1, hVps24, and hVps20 shown here also known as CHMP4a, CHMP3, and CHMP6, respectively. The myristoylation site in Vps20 [8] is highlighted in red. The MIM2 motif in CHMP6 [6] is highlighted in green. The putative MIM2 of Nmar_0816 is highlighted in blue. The multiple sequence alignment was performed using ClustalW2 program.

Figure S6

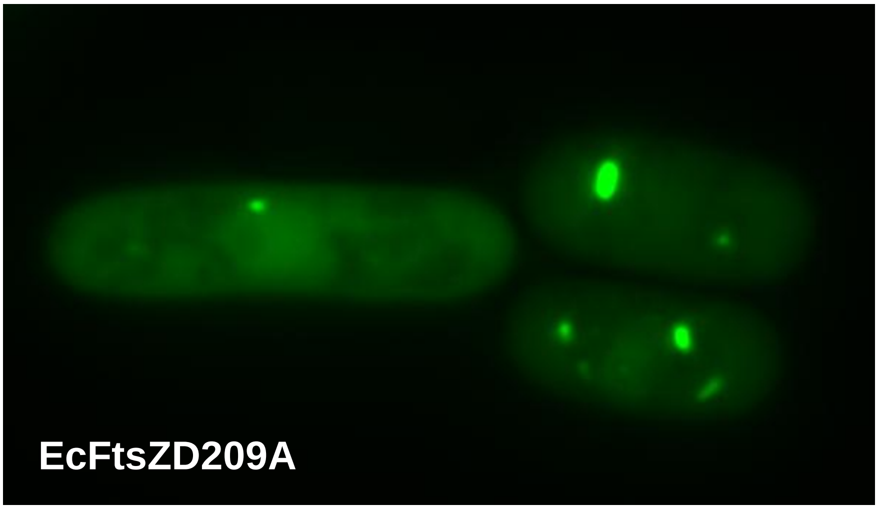

Figure S6: Expression of *E. coli* FtsZ mutant (D209A) in yeast.
Image of yeast cells expressing *E. coli* FtsZ-GFP carrying a mutation at T7 loop (EcFtsZD209A).
