# Supplementary material for: The Nitrosopumilus maritimus CdvB, but Not FtsZ, Assembles into Polymers
Source: Archaea. 2013 Jun 2;2013:104147. doi: 10.1155/2013/104147 (PMC3684127; doi:10.1155/2013/104147)
Supplement: Supplementary file 1 — The Supplemental information includes 2 videos (formation of Nmar_0816-GFP filaments in yeast (Video S1); yeast cell death caused by failure to dissolve Nmar_0816 polymers during cell division (Video S2)), 6 figures (multiple sequence alignment of the CdvB genes from N. maritimus and S. acidocaldarius (Figure S1); diverse polymeric structures formed by Nmar_0816 in yeast (Figure S2); yeast cell death caused by failure to dissolve Nmar_0816 polymers (Figure S3); localization of Nmar_0816 to the rim of endosomes in NRK cells (Figure S4); multiple sequence alignment of the CdvB genes from N. maritimus and ESCRT-III genes from human and fission yeast (Figure S5); expression of E. coli FtsZ mutant (D209A) in yeast (Figure S6)), and 2 tables (list of primers used in this study (Table S1); list of yeast strains used in this study (Table S2)). [file 104147.f1.zip › Supplementary tables.pdf]

Table S1

Primer	Sequence	Gene/Construct
MOH4953	CTGACTTATAGTCGCTTTGTTAAATCATATGGCTAATCTTTCTAACAAATGG	Nmar_0029
MOH4954	AGCGTTGTTGTTGTTCCCGGGATCCCCATAAAACTTTGATGTAGATTC	Nmar_0029
MOH4955	CTGACTTATAGTCGCTTTGTTAAATCATATGCCTAATTTTGATAAAACTTGG	Nmar_0061
MOH4956	AGCGTTGTTGTTGTTCCCGGGATCCCCTTCAAATGTTGAAGTAGATGT	Nmar_0061
MOH4957	CTGACTTATAGTCGCTTTGTTAAATCATATGTTATCTAATTCATGGAATAAC	Nmar_0816
MOH4958	AGCGTTGTTGTTGTTCCCGGGATCCCCGATAAAAATATCAGTTTTCTT	Nmar_0816
MOH5004	CTGACTTATAGTCGCTTTGTTAAATCATATGTCTATGGCACCTCAAGAATTG	Nmar_1088
MOH5005	AGCGTTGTTGTTGTTCCCGGGATCCCCAAGAGCTTGAAATTCTTCAGAC	Nmar_1088
MOH4951	CTGACTTATAGTCGCTTTGTTAAATCATATGTCTTTTCAAGTTAAAGAACCT	Nmar_1262
MOH4952	AGCGTTGTTGTTGTTCCCGGGATCCCCTTCTAATTGGTAAAGATCAAG	Nmar_1262
MOH5101	GGTGGAGGTACTGGAACTGGTATGGCACCTGTTGTTTCAGAAATG	Nmar_1262**
MOH5102	ACCAGTTCCAGTACCTCCACCAAGATTAGACATCATAACAATAG	Nmar_1262**
MOH5155	CTGACTTATAGTCGCTTTGTTAAATCATATGACTGTTTCTGAATTGGGAGATG	Nmar_0816$_{(109\text{-}216)}$
MOH5156	AGCGTTGTTGTTGTTCCCGGGATCCCCATCAAGTCTTAACTTAACCTGTTC	Nmar_0816$_{(1\text{-}108)}$
MOH5294	AGCGTTGTTGTTGTTCCCGGGATCCCCAGTTTGTCCTGCAATAACACCTTG	Nmar_0816$_{(1\text{-}192)}$
MOH4981	GTTGTTGTTACACTTTCTCCTGCTATGTCAATTATTAAGGGTCTTG	Nmar_0816$_{C123A}$
MOH4982	CAAGACCCTTAATAATTGACATAGCAGGAGAAAGTGTAACAACAAC	Nmar_0816$_{C123A}$
MOH4964	CCCCCCGAATTCATGTTATCTAATTCATGGAAT	Nmar_0816 in pEGFP
MOH4963	CCCCCCCCCGGGTTAGATAAAAATATCAGTTTTC	Nmar_0816 in pEGFP
MOH4962	CCCCCCGGATCCATGTTATCTAATTCATGGAAT	Nmar_0816 in pQE30
MOH4963	CCCCCCCCCGGGTTAGATAAAAATATCAGTTTTC	Nmar_0816 in pQE30

Table S1: List of primers used in this study.

Table S2

Strain	Genotype	Source
1	*ura4*-D18 *leu1*-32 h- (MBY192)	Lab collection
2	*nmt42*-GFP::*ura4*$^{+}$ *ura4*-D18 *leu1*-32 h- (MBY3495)	Lab collection
3	*nmt42*-Nmar_0029-GFP::*ura4*$^{+}$ *ura4*-D18 *leu1*-32 h-	This study
4	*nmt42*-Nmar_0061-GFP::*ura4*$^{+}$ *ura4*-D18 *leu1*-32 h-	This study
5	*nmt42*-Nmar_0816-GFP::*ura4*$^{+}$ *ura4*-D18 *leu1*-32 h-	This study
6	*nmt42*-Nmar_1088-GFP::*ura4*$^{+}$ *ura4*-D18 *leu1*-32 h-	This study
7	*nmt42*-Nmar_0816-GFP::*ura4*$^{+}$ *ura4*-D18 *leu1*-32 h- (RE)	This study
8	*nmt42*-Nmar_0816$_{(1\text{-}108)}$-GFP::*ura4*$^{+}$ *ura4*-D18 *leu1*-32 h-	This study
9	*nmt42*-Nmar_0816$_{(109\text{-}216)}$-GFP::*ura4*$^{+}$ *ura4*-D18 *leu1*-32 h-	This study
10	*nmt42*-Nmar_0816$_{(1\text{-}192)}$-GFP::*ura4*$^{+}$ *ura4*-D18 *leu1*-32 h-	This study
11	*nmt42*-Nmar_0816$_{C123A}$-GFP::*ura4*$^{+}$ *ura4*-D18 *leu1*-32 h-	This study
12	*nmt42*-Nmar_1262-GFP::*ura4*$^{+}$ *ura4*-D18 *leu1*-32 h-	This study
13	*nmt42*-Nmar_1262**-GFP::*ura4*$^{+}$ *ura4*-D18 *leu1*-32 h-	This study
14	*nmt42*-EcFtsZ-GFP::*ura4*$^{+}$ *ura4*-D18 *leu1*-32 h- (MBY3497)	Lab collection
15	*nmt42*-EcFtsZD209A-GFP::*ura4*$^{+}$ *ura4*-D18 *leu1*-32 h- (MBY4742)	Lab collection

RE: Yeast strain constructed using plasmid cloned by restriction-based method.
MBY: Lab strains.

Table S2: List of yeast strains used in this study.
